# Awareness gaps of onchocerciasis in endemic Health district of Kéniéba, Séféto, Dioila, Kati and Selingué in 2021: cross - sectional study in Mali

**DOI:** 10.3389/fpubh.2026.1858711

**Published:** 2026-07-22

**Authors:** Housseini Dolo, Yaya Ibrahim Coulibaly, Mahamoud Mahamadou Koureichi, Yacouba Sangaré, Boubacar Guindo, Mama Niele Doumbia, Modibo Keita, Moussa Sangare, Siaka Yamoussa Coulibaly, Salif Seriba Doumbia, Seydou Doumbia, Thomas B. Nutman

**Affiliations:** 1 Laboratory of Public Health, Faculty of Medicine and Odontostomatology, University of Sciences, Techniques and Technologies of Bamako, Bamako, Mali; 2 Neglected Tropical Diseases Training and Research Unit, International Center of Excellence in Research, Bamako, Mali; 3 National Onchocerciasis Control Program, General Directorate for Health and Public Hygiene, Bamako, Mali; 4 Helen Keller International, Bamako, Mali; 5 Laboratory of Parasitic Diseases, National Institute of Health, Bethesda, MD, United States

**Keywords:** onchocerciasis, awareness, endemic health district, elimination, rural population, Mali

## Abstract

**Background:**

Onchocerciasis is still endemic and requiring Mass drug administration (MDA). In Mali, despite more than 15 rounds of treatment, community perception and adherence depend critically on awareness of the disease. This study aimed to assess awareness and knowledge gaps in the context of long-term MDA exposure assess in endemic communities receiving ivermectin MDA.

**Methods:**

A cross-sectional survey was conducted in the health districts (HD) of Dioila, Kati, Kéniéba, Séféto and Sélingué in Mali after 2020 ivermectin MDA distribution. The survey involved 12,663 individuals from 2,357 households. Data on knowledge, attitudes, and reasons for drug acceptance were collected using the ODK platform and analyzed with SPSS.

**Results:**

Among those who swallowed ivermectin, the primary reasons were to avoid contracting the disease 45.2% (2,689/5952) or to stay healthy 44.0% (2,619/5952), with significant differences across districts (*p* < 0.001). Awareness of onchocerciasis was high overall (95.6%), ranging from 89% in Kéniéba to 99.6% in Sélingué (*p* < 0.001). However, knowledge of the mode of transmission was poor: only 32.1% (724/2254) knew it was transmitted by black flies, with the lowest rates in Kati (19.6%) and Sélingué (20.9%). Knowledge of the existence of treatment varied from 45.3% in Kati to 96.2% in Sélingué (*p* < 0.001), but among those who aware, correct identification of ivermectin as the treatment was high (96.9%).

**Conclusion:**

While awareness of onchocerciasis as a disease is high in most districts after 15 + MDA rounds, critical knowledge gaps persist regarding transmission and treatment purpose. Many individuals take ivermectin for general health or due to instruction rather than specific prevention. Targeted health education to explain transmission and the rationale for MDA is needed to sustain adherence and progress toward elimination.

## Introduction

Onchocerciasis due to *Onchocerca volvulus* is transmitted from person to person by repeated bites of infected black flies, mainly in tropical areas (1). The number of people infected with onchocerciasis is estimated at 37 million in 36 countries, 99% of whom live in 31 countries in sub-Saharan Africa. Approximately 500,000 of these people suffer significant vision loss and half are blind (2). The number of people at risk of onchocerciasis infection is estimated at 85.6 million in the world (3).

In Mali, the public health importance of onchocerciasis was reported as early as the 1970s (4). Onchocerciasis was initially considered endemic in five regions in Kayes, Koulikoro, Sikasso, Ségou and Mopti. According to the Mali National onchocerciasis control program (PNLO in French), 34 health districts out of the 75 in the country were endemic for onchocerciasis between 1971 and 1987 with a total of 4,589,963 inhabitants at risk of onchocerciasis (5).

Before control interventions (vector and mass drug administration), onchocerciasis prevalence varied notably across the five health districts. Kéniéba had the highest levels, typically exceeding 45–60%, while Séféto showed high prevalence ranging from about 30 to 60%. Sélingué presented intermediate values, generally between 15 and 50%, and Dioïla had moderate prevalence around 15–30%. In contrast, Kati showed the lowest levels, with prevalence usually between less than 5 and 15% (National onchocerciasis control data).

The PNLO was established in 1986 to control the disease as a public health problem. In 1987 the distribution of ivermectin was initiated, donated by Merck & Co. and later with community-directed ivermectin treatment (CDTI) with the support of the African Program for Onchocerciasis Control (APOC) and using community drug distributors (CDD).

Each year, for more than 25 years, the PNLO has conducted mass drug administration (MDA) campaigns in 20 onchocerciasis-endemic health districts.

Several observations that may affect the quality of coverage rates have emerged from supervision visits during MDA campaigns and from other studies on factors associated with the use of drugs against neglected tropical diseases (NTDs) (5, 6). The observations were the non-compliance with the targets to be treated; inadequate towers; drug shortages; non-use of advice cards; under-information of communities on the campaign periods; data collection issues; and lack of knowledge of NTDs.

To assess the awareness of onchocerciasis, we embedded a knowledge, attitudes and practices survey in a treatment coverage survey conducted by national onchocerciasis control program few months after the campaign as recommended by the World Health Organization (WHO) in the health districts of Diola, Kati, Kénièba, Séféto, and Sélingué in Mali to study the knowledge of onchocerciasis and its treatment.

## Methods

### Study design

The study was a cross-sectional survey conducted between December 2020 and January 2021. It was implemented shortly after the completion of the 2020 mass drug administration (MDA) campaign as part of a routine coverage evaluation survey recommended by the World Health Organization. The timing of the data collection allowed for the assessment of participants’ knowledge, perceptions, and practices related to onchocerciasis and ivermectin uptake in the immediate post-MDA period.

### Study sites

**Figure 1:**
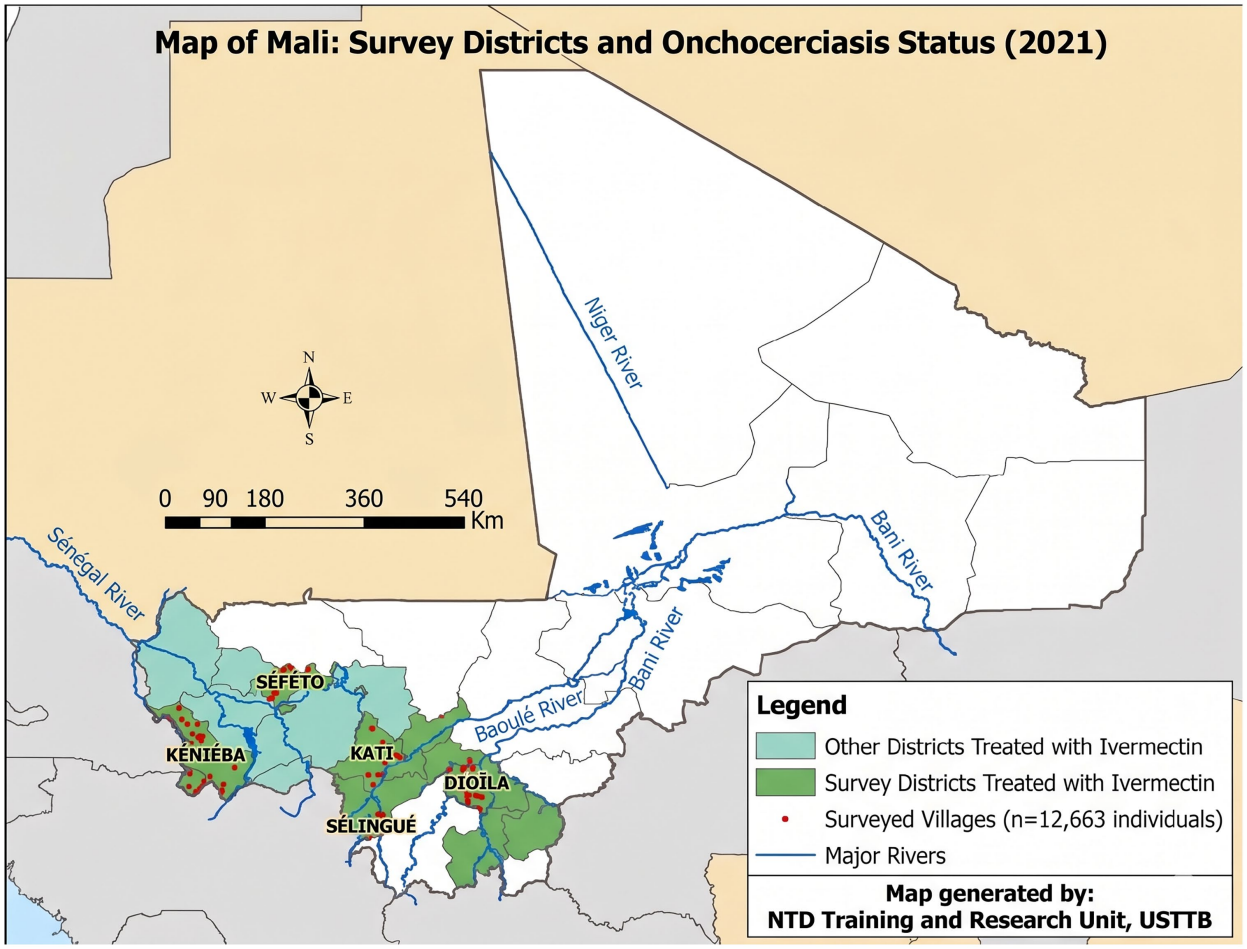
Map of Mali showing the surveyed villages.

This study was conducted in the health districts of Kéniéba, Séféto, Dioila, Kati, and Sélingué (Figure 1). These health districts were chosen because of the implementation of the MDA.

### Study population

All individuals aged 5 years and above who were present in selected households during the survey period were eligible to participate and were directly interviewed. For household members who were absent at the time of the survey, information was obtained from the head of household or another adult caregiver, in accordance with standard World Health Organization coverage survey procedures. For children who were unable to reliably respond to the questionnaire, typically those under 10 years of age, information was provided by their parents or caregivers.

### Sampling

For the ivermectin MDA coverage survey, the Coverage Survey Builder (CSB) software package was used to conduct a three-stage sampling (7).

#### Primary sampling unit

While using the systematic random sampling technique, we selected two (02) districts in the Kayes region, two (02) in the Koulikoro region and one (01) in the Sikasso region. This random selection process was conducted by the PNLO (National Onchocerciasis Control Program). To know the name of the drawn districts, we apply the corresponding number chosen.

#### Secondary sampling unit

The second stage of sampling involved identifying segments within each village. For each district, a list of administratively recognized villages was compiled using updated population data from 2020. The population per village was divided by six (the average number of individuals per household in Mali) to estimate the number of households. A segment was defined as a group of 50 households. Villages with fewer than 25 households were paired with a neighboring village. The adjusted number of households was then entered into the Coverage Survey Builder (CSB) software.

Depending on the population size, a village could comprise one or more segments. Thirty (30) segments per district were selected using randomization procedures generated by the CSB, in line with recommended methodology. The role of village or community chiefs was limited to facilitating the identification and access to the randomly selected segments, rather than conducting or influencing the selection process.

If a selected village or segment was inaccessible due to geographical barriers (long distances or poor road conditions) or security constraints, a replacement segment was selected within the same health area following the same sampling procedures.

#### Tertiary sampling unit

The third stage of sampling involved identifying the households to be surveyed within each selected segment. A comprehensive list of households was established for each segment using existing village registers where available. In cases where such records were incomplete or unavailable, survey teams conducted direct household enumeration with the assistance of local leaders. This ensured a complete and up-to-date sampling frame prior to household selection.

From this list, households were selected according to the Coverage Survey Builder (CSB) procedure (A or B list). In each selected household, all eligible individuals were enrolled. Based on CSB assumptions, an average of four individuals per household (approximately 80% of the total household size, estimated at six persons) were expected to be included.

Selected households were not replaced if residents were absent at the time of the visit. To minimize non-response, survey teams revisited households before leaving the village or segment on the same day. Data collection within each segment continued until 15 households were surveyed. If the target number of individuals per segment (60) was reached before completing 15 households, the survey continued until all selected households were included. Conversely, if 15 households were completed before reaching 60 individuals, the survey ended at that point.

### Selection of participants

The fourth level corresponded to the statistical unit, represented by the individual participant. In each selected household, the structured questionnaire was administered to all individuals who were present at the time of the survey and met the eligibility criteria. For children who could not reliably answer the questions, responses were provided by their parents or legal guardians.

After completing data collection in the first household, survey teams proceeded sequentially to the next selected household within the segment. This process continued until the required 15 households per segment had been surveyed.

### Sample size calculation

For the community surveys, sampling principles recommended by the World Health Organization (WHO) and implemented through the Coverage Survey Builder (CSB) were used to determine the number of segments, sample size, and number of households to be surveyed per segment (8). For each district, the required sample size was calculated using CSB based on the following parameters: expected coverage rate (50%), precision (5%), design effect (4.0), alpha risk (5%), and a non-response rate of 15%. Additional parameters included the assumed number of individuals per household (4.0), target segment size (50 households), and the number of clusters selected (30).

Based on these assumptions, the estimated total sample size was 9,040 individuals (1,808 per district across five districts). The sampling interval between households was 3.31, and approximately 15 households were targeted per segment. According to CSB calculations, the final sample size per health district was 1,808 individuals.

### Study instrument

A structured questionnaire was used to collect data from households through the Open Data Kit (ODK) platform using smartphones. The questionnaire covered key domains including socio-demographic characteristics, ivermectin intake, information on the mass drug administration campaign, and knowledge of onchocerciasis.

The instrument was developed based on WHO guidelines for coverage surveys and Knowledge, Attitudes, and Practices (KAP) assessments and was adapted to the Malian context. Prior to implementation, the questionnaire was reviewed by experts from the National Onchocerciasis Control Program and the research team to ensure content validity. It was subsequently piloted in a non-study area, and minor modifications were made to improve clarity and cultural relevance.

The use of the ODK platform helped standardize data collection procedures and minimize inconsistencies. However, formal psychometric validation (e.g., assessment of internal consistency such as Cronbach’s alpha) was not conducted.

### Data collection

Data were collected by trained field survey teams. Training covered questionnaire administration, use of the Open Data Kit (ODK) platform, and ethical procedures, including informed consent and confidentiality. Field supervisors were responsible for overseeing data collection, ensuring adherence to the study protocol, and conducting routine quality control checks to maintain data accuracy and completeness.

### Data analysis

Socio-demographic characteristics, including age, sex, village of residence, schooling status, level of education, type of school attended, main occupation, and marital status, were described. Knowledge, attitudes, and practices (KAP) variables included knowledge of transmission modes, prevention methods, signs of the disease, treatment, consequences, and awareness of mass drug administration targeting onchocerciasis. Analyses also examined reasons for acceptance or refusal of treatment, as well as sources of information. Associations between age and reasons for non-treatment were explored. Assessment of knowledge about onchocerciasis was based on responses provided either by the participant or, when applicable, by the head of the household.

Statistical analyses were performed using the Statistical Package for Social Sciences (SPSS) version 27.0. For bivariate analysis, Pearson’s Chi-square test (or Fisher’s exact test, when appropriate) was used to compare proportions, with a significance level set at *α* = 5%. The reported *p*-values are intended to highlight patterns and potential gaps across districts rather than to establish definitive inferential conclusions. As this study is descriptive in nature, *p*-values were used as a screening tool to identify differences in KAP indicators across districts. No adjustments for multiple comparisons were performed, and findings should therefore be interpreted with caution.

### Ethical considerations

The protocol was approved by the Ethics Committee of the University of Sciences, Techniques, and Technologies of Bamako (USTTB) under approval number 2021/231/USTTB. In the field, participation was based on oral informed consent obtained at the household level, as approved by the ethics committee. Prior to data collection, survey teams provided participants with information about the study, including its context, objectives, and procedures. Individuals were then asked whether they agreed to participate, and only those who consented were included in the survey.

For participants aged 5 to 17 years, consent was obtained from parents or legal guardians. In addition, children who were able to understand the study were asked for their willingness to participate, and interviews were conducted only if they agreed. For younger children who were unable to reliably respond, information was provided by their caregivers.

To ensure confidentiality, no identifying information linking participants to their responses was collected or reported.

## Results

### Treatment adherence

The survey involved 12,663 individuals from 2,357 households (Table 1). Those who swallowed the drugs did so to avoid contracting the disease or to be healthy, with 45.2% (2,689/5952) and 44.0% (2,619/5952) respectively. The difference was statistically significant between the health districts for all variables considered (Table 2).

**Table 1:** Sociodemographic characteristics of respondents by health district.

Parameters	Health district						*p*
	Dioila	Kati	Kéniéba	Séféto	Sélingué	Total	
	*N* = 2,573	*N* = 2,174	*N* = 2,647	*N* = 2,764	*N* = 2,505	*N* = 12,663	
	*n* (%)	*n* (%)	*n* (%)	*n* (%)	*n* (%)	*n* (%)	
Status of respondents by survey acceptance							
Number surveyed	2,565 (99.7)	2,160 (99.4)	2,624 (99.1)	2,762 (99.9)	2,488 (99.3)	12,599 (99.5)	**0.0002**
Number of refusals	8 (0.3)	14 (0.6)	23 (0.9)	2 (0.1)	17 (0.7)	64 (0.5)	
Gender							
Male	1,310 (51.1)	1,116 (51.7)	1,286 (49.0)	1,392 (50.4)	1,286 (51.7)	6,390 (50.7)	0.29
Female	1,255 (48.9)	1,044 (48.3)	1,338 (51.0)	1,370 (49.6)	1,202 (48.3)	6,209 (49.3)	
Age group							
<5 years	360 (14.0)	374 (17.3)	442 (16.8)	473 (17.1)	449 (18.0)	2098 (16.7)	**0.0005**
5–14 years	815 (31.8)	585 (27.1)	769 (29.3)	849 (30.7)	732 (29.4)	3,750 (29.8)	
15 years and above	1,390 (54.2)	1,201 (55.6)	1,413 (53.8)	1,440 (52.1)	1,307 (52.5)	6,751 (53.6)	
Level of education	*N* = 2,392	*N* = 1994	*N* = 2,401	*N* = 2,207	*N* = 2,179	*N* = 11,173	**0.0001**
None schooled	1,377 (57.5)	867 (43.5)	1,589 (66.2)	1,407 (63.75)	1,203 (55.3)	6,443 (57.7)	
Schooled	1,015 (42.5)	1,127 (56.5)	812 (33.8)	800 (36.2)	976 (44.8)	4,730 (42.4)	

Bold values indicate significant *p* values.

**Table 2:** Distribution of respondents who swallowed ivermectin by reason for taking the drug during the coverage survey in 2021.

Reason	District sanitaire						*p*
	Dioila	Kati	Kénièba	Séféto	Sélingué	Total	
	*N* = 1888	*N* = 662	*N* = 884	*N* = 997	*N* = 1,521	*N* = 5,952	
	*n* (%)	*n* (%)	*n* (%)	*n* (%)	*n* (%)	*n* (%)	
Fear of contracting the disease	1,267 (67.1)	391 (59.1)	110 (12.4)	228 (22.9)	693 (45.6)	2,689 (45.2)	**0.001**
Being healthy	462 (24.5)	119 (18.0)	553 (62.6)	697 (69.9)	788 (51.8)	2,619 (44.0)	**0.001**
I was instructed to swallow	9 (0.5)	111 (16.8)	116 (13.1)	29 (2.9)	2 (0.1)	267 (4.5)	**0.001**
Against onchocerciasis	115 (6.0)	17 (2.6)	29 (3.3)	1 (0.1)	7 (0.5)	169 (2.8)	**0.001**
Everyone takes it	14 (0.7)	13 (2.0)	52 (5.9)	11 (1.1)	7 (0.5)	97 (1.6)	**0.001**
Drug is effective	20 (1.1)	4 (0.6)	1 (0.1)	22 (2.2)	4 (0.3)	51 (0.9)	**0.001**
Someone told me about it	0 (0.0)	3 (0.5)	23 (2.6)	6 (0.6)	9 (0.6)	41 (0.7)	**0.001**
Do not know	1 (0.1)	4 (0.6)	0 (0.0)	3 (0.3)	11 (0.7)	19 (0.3)	–

Pearson’s Chi-square test; Fisher’s exact test. Bold values indicate significant *p* values.

### Knowledge and perceptions

Among the respondents, those who said they had heard of onchocerciasis represented 99.1% (465/469) in Dioila, 94.9% (424/447) in Kati, 89% (430/483) in Kénièba, 95.7% (466/487) in Séféto and 99.6% (469/471) in Sélingué. This rate of knowledge of onchocerciasis was statistically different between the health districts (*p* < 0.001). For the presence of *simulium*, the respondents said that these vectors were present in their village with the following proportions: 94.2% (442/469) in Dioila, 56.2% (251/447) in Kati, 80.3% (388/483) in Kéniéba, 92.6% (451/487) in Séféto and 79.4% (374/471) in Sélingué with a statistically significant difference between the health districts (*p* < 0.001). Regarding knowledge of the existence of treatment for onchocerciasis, this varied from 86.2% (401/465) in Dioila, 45.3% (192/424) in Kati, 69.3% (298/430) in Kénièba, 68.9% (321/466) in Séféto, and 96.2% (451/469) in Sélingué (*p* < 0.001). For the type of treatment mentioned, it was correct when talking about ivermectin in the following proportions: 99.3% (398/401) in Dioila, 85.4% (164/192) in Kati, 94.6% (282/298) in Kénièba, 98.8% (317/321) in Séféto and 100% (451/451) in Sélingué (*p* < 0.001). Regarding knowledge of the mode of transmission of onchocerciasis, the proportions were 32.3% (150/465) in Dioila, 19.6% (83/424) in Kati, 49.1% (211/430) in Kénièba, 39.1% (182/466) in Séféto and 20.9% (98/469) in Sélingué (*p* < 0.001) (Table 3).

**Table 3:** Knowledge, attitudes and practices regarding onchocerciasis in the health districts of Kéniéba, Séféto, Dioila, Kati, and Sélingué in 2021.

KAP for onchocerciasis	Health district						*p*
	Dioila	Kati	Kénièba	Séféto	Sélingué	Total	
	*n* (%)	*n* (%)	*n* (%)	*n* (%)	*n* (%)	*n* (%)	
Heard about onchocerciasis							
No	4 (9)	23 (5.1)	53 (11)	21 (4.3)	2 (0.4)	103 (4.4)	**0.001**
Yes	465 (99.1)	424 (94.9)	430 (89)	466 (95.7)	469 (99.6)	2,254 (95.6)	
Total	469 (100)	447 (100)	483 (100)	487 (100)	471 (100)	2,357 (100)	
Presence of Black flies in your village							
No	22 (4.7)	172 (38.5)	89 (18.4)	31 (6.4)	27 (5.7)	341 (14.5)	**0.001**
Yes	442 (94.2)	251 (56.2)	388 (80.3)	451 (92.6)	374 (79.4)	1906 (80.9)	
Do not know	5 (1.1)	24 (5.4)	6 (1.2)	5 (1)	70 (14.9)	110 (4.7)	
Total	469 (100)	447 (100)	483 (100)	487 (100)	471 (100)	2,357 (100)	
Existence of treatment/onchocerciasis							
No	34 (7.3)	161 (38)	82 (19.1)	118 (25.3)	12 (2.6)	407 (18.1)	**0.001**
Yes	401 (86.2)	192 (45.3)	298 (69.3)	321 (68.9)	451 (96.2)	1,663 (73.8)	
Do not know	30 (6.5)	71 (16.7)	50 (11.6)	27 (5.8)	6 (1.3)	184 (8.2)	
Total	465 (100)	424 (100)	430 (100)	466 (100)	469 (100)	2,254 (100)	
Type of treatment							
Ivermectin	398 (99.3)	165 (85.4)	282 (94.6)	317 (98.8)	451 (100)	1,613 (96.9)	**0.001**
Traditional medicines	3 (0.7)	25 (13)	16 (5.4)	4 (1.2)	0 (0)	48 (2.9)	
Vaccines	0 (0)	2 (1)	0 (0)	0 (0)	0 (0)	2 (0.1)	
Total	401 (100)	192 (100)	298 (100)	321 (100)	451 (100)	1,663 (100)	
Onchocerciasis transmission modes							
No	315 (67.7)	341 (80.4)	219 (50.9)	284 (60.9)	371 (79.1)	1,530 (67.9)	**0.001**
Yes	150 (32.3)	83 (19.6)	211 (49.1)	182 (39.1)	98 (20.9)	724 (32.1)	
Total	465 (100)	424 (100)	430 (100)	466 (100)	469 (100)	2,254 (100)	

KAP, knowledge, attitudes and practices; Pearson’s Chi-square test; Fisher’s exact test. Bold values indicate significant *p* values.

## Discussion

Our findings reveal a paradoxical situation in Mali’s onchocerciasis-endemic districts. After more than 15 rounds of mass drug administration (MDA), while over 89% of respondents had heard of the disease, profound gaps persist in understanding its transmission and the specific rationale for treatment. This disconnect between general disease awareness and biomedical comprehension is not unique to Mali but reflects challenges documented across sub-Saharan Africa.

The most critical gap identified in our study is the low correct knowledge of transmission (32.1% overall), which is strikingly similar to findings from Ogun State, Nigeria, where only 35.9% of community members correctly identified blackfly bites as the cause of onchocerciasis (9). In the Democratic Republic of Congo, despite 96.9% of respondents knowing about the disease, only 49.9% correctly named the blackfly as the vector (10). Our results from Kati (19.6%) and Sélingué (20.9%) fall at the lower end of this spectrum, suggesting that even districts with long-standing MDA programs have not effectively conveyed this fundamental epidemiological concept.

Our observation that 44% of respondents took ivermectin “to be healthy” rather than specifically “to avoid contracting the disease” (45.2%) aligns with the literature documenting that communities often perceive ivermectin as a general “health tonic” or broad-spectrum antiparasitic. This is particularly evident in our data from Kéniéba and Séféto, where “being healthy” was the dominant reason (>62%). Similar patterns have been reported from Ethiopia, where only 35.4% of community drug distributors (CDDs) had good knowledge about onchocerciasis, and from Cameroon, where communities were not adequately involved in deciding distribution times and modes (11, 12).

The wide inter-district variation we observed from 45.3% knowledge of treatment existence in Kati to 96.2% in Sélingué likely reflects differences in the intensity and quality of health education, the duration of active MDA, and possibly local epidemiological factors such as proximity to Simulium breeding sites. Sélingué’s high performance (100% correct naming of ivermectin among those aware) may serve as a best-practice model, potentially benefiting from more consistent messaging or a higher perceived burden of skin disease. Conversely, Kati’s low scores are concerning given its peri-urban character, where MDA implementation is often more challenging due to population mobility and competing health priorities (13, 14).

These awareness gaps are not merely academic. The WHO 2021–2030 roadmap for neglected tropical diseases explicitly calls for enhanced community engagement and education to achieve onchocerciasis elimination (15). Our findings indicate that current MDA campaigns in Mali may be over-reliant on drug distribution alone, without sufficient investment in knowledge transfer. When communities do not understand that ivermectin interrupts transmission by reducing skin microfilariae available to blackflies, they may disengage once immediate symptoms (e.g., itching) subside. This directly threatens the sustained high coverage (>80%) required for elimination (16).

Furthermore, the persistent uncertainty we observed even among those who swallow the drug echoes the Guatemala experience, where 3 years after halting MDA, 53% of community members remained unsure that onchocerciasis had truly been eliminated, and 63% wanted to continue receiving ivermectin (17). That study demonstrated that without adequate knowledge, post-elimination surveillance and community buy-in are compromised.

## Strengths and limitations

This study has several strengths, including a large sample size (*N* = 12,663), the use of a rigorous WHO-recommended three-stage sampling approach implemented through Coverage Survey Builder software, and the inclusion of five districts representing diverse epidemiological and programmatic contexts. These factors enhance the generalizability of the findings across onchocerciasis-endemic areas in Mali.

However, several limitations should be acknowledged. First, the cross-sectional design precludes establishing causal relationships between knowledge levels and long-term treatment adherence. Second, social desirability bias may have led to an overestimation of reported knowledge and reasons for drug acceptance, as respondents may have provided answers they perceived as favorable to interviewers.

In addition, some information was obtained through proxy respondents, particularly for household members who were absent at the time of the survey and for younger children who were unable to respond. This may have introduced recall and proxy-reporting biases, as second-hand responses may not accurately reflect the true knowledge or perceptions of those individuals.

Finally, although the questionnaire was developed based on WHO guidelines and reviewed by experts, it did not undergo formal psychometric validation. In particular, internal consistency measures (e.g., Cronbach’s alpha) were not assessed, which may affect the reliability of the knowledge, attitudes, and practices indicators.

### Programmatic recommendations

Based on our findings and the broader literature, we recommend that Mali’s National Onchocerciasis Control Program (PNLO) implement several targeted actions. First, health education modules should be developed that explicitly address the role of blackflies as the vector, how ivermectin works to interrupt transmission, and the importance of taking the drug even when asymptomatic. These messages must be delivered in local languages (Bambara, Malinké, Fulfuldé, etc.) and adapted to district-specific knowledge gaps. Second, enhanced training for community drug distributors (CDDs) is essential, as studies from Ethiopia and Cameroon have shown that CDD knowledge is often inadequate (11, 12). This training should include not only drug distribution logistics but also communication skills, the use of visual aids, and techniques for addressing local misconceptions. Third, multichannel communication strategies should be employed, utilizing community radio, religious gatherings, schools, and mobile health messages. Finally, regular knowledge, attitudes, and practices (KAP) assessments should be integrated into MDA monitoring frameworks, as recommended by the WHO, to identify persistent gaps and guide adaptive programming.

## Conclusion

In conclusion, while Mali has achieved remarkable progress in sustaining MDA for onchocerciasis over more than 15 years, our study demonstrates that awareness of transmission mechanisms and treatment rationale remains dangerously low in several endemic districts. Closing these knowledge gaps is not a luxury but a necessity for achieving the WHO 2030 elimination targets. Future investments must balance drug distribution with community-centered health education that transforms superficial awareness into genuine understanding and sustained behavior change.

## Data Availability

The original contributions presented in the study are included in the article/supplementary material, further inquiries can be directed to the corresponding author.
